# Albert V. D. Collier

**Published:** 1913-04

**Authors:** 


Albert V. D. Collier, Albany, 1866, died in Albany December 15, 1912, aged 75. He was a resident of Catskill.

